# Influence of Mechanical Grinding on Particle Characteristics of Coal Gasification Slag

**DOI:** 10.3390/ma15176033

**Published:** 2022-09-01

**Authors:** Mengbo Zhu, Geng Xie, Lang Liu, Pan Yang, Huisheng Qu, Caixin Zhang

**Affiliations:** 1 College of Energy Engineering, Xi’an University of Science and Technology, Xi’an 710054, China; 2 Research Center for Functional Backfill Technology in Mine, Xi’an University of Science and Technology, Xi’an 710054, China; 3 Key Laboratory of Western Mines and Hazards Prevention, Ministry of Education of China, Xi’an 710054, China

**Keywords:** coal gasification slag, particle size distribution, grinding kinetics, strength activity index, grey relational analysis

## Abstract

Based on the test results of laser particle size analyzer, specific surface area analyzer and infrared spectrometer, the grinding kinetics of coal gasification slag (CGS) was systematically described by using Divas–Aliavden grinding kinetics, Rosin–Rammler–Bennet (RRB) distribution model and particle size fractal theory. The influence of grinding time and particle group of CGS on the strength activity index of mortar was studied by using the strength activity index of mortar and grey correlation analysis. The results show that the particles are gradually refined before mechanical grinding of CGS for 75 min. When the mechanical grinding time is greater than 75 min, the “agglomeration phenomenon” of fine CGS particles led to the decrease in various properties. Divas–Aliavden grinding kinetics, the RRB model and fractal dimension can characterize the change of CGS particle size in the grinding process quantitatively. The strength activity index of CGS at different curing ages is positively correlated with grinding time, and the influence on the later strength activity index is the most obvious. The relationship between CGS particle size distribution and strength activity index were probed using grey correlation analysis. The CGS particle groups with the particle size of 20~30 μm and 10~20 μm have the greatest impact on the early and late strength activity index, respectively. Therefore, the optimal grinding time of CGS as auxiliary cementing material is 75 min, considering factors, such as economy and performance, and the specific surface area (SSA) is 4.4874 m^2^·g^−1^.

## 1. Introduction

The goal of carbon peak and carbon neutralization is related to the community of human and nature. However, restricted by the resource system of “rich coal, poor oil and less gas” in China, coal resources are still in the core position of resource utilization. Therefore, in this era where environmental issues have attracted much attention, it is an inevitable trend to vigorously promote the development of technologies related to the clean utilization of coal, and coal gasification technology as a means of clean utilization of coal has gradually matured [1]. Coal gasification refers to the process in which coal reacts with gasification agent at high temperatures to produce gas products (CO, CH_4_, H_2_, etc.) and coal gasification slag (CGS) [2,3]. The CGS generated by air flow gasification can be divided into coarse and fine slags. Coarse slags are mainly composed of glass phase pellets and tightly packed solids, presenting a lamellar structure on a macroscopic scale and containing more active substances of volcanic ash. Fine slag, where the particle size is less than 350 μm, is taken out the gasifier by gas products. Compared with coarse slag, fine slag contains more carbon residue, which is porous and loose and restricts the alkali–silica reaction [4]. Our study focuses on coarse slag, which contains potential cementitious activities. Compared with the traditional coal combustion and utilization, the environmental pollution caused by coal gasification is relatively low, but a large amount of CGS accumulation will cause serious damage to the originally ecologically fragile parts of northern Shaanxi in China [5,6]. According to the investigation of the Yulin Bureau of Statistics, about 32 million tons of CGS were produced in 2016, of which about 12.5% were produced in Yulin City, Shaanxi Province. It is expected that the production of CGS in Yulin City will reach 10.3 million tons in 2023, and the need of treatment for CGS as a solid waste is imminent.

The research results of domestic and foreign scholars on CGS show that the main chemical composition of CGS is similar to that of Portland cement, and the internal mineral phase is mainly amorphous glass phase. Related research shows that it has certain cementitious activity [7,8,9,10]. For example, the research of Sheng et al. [11] shows that a certain fineness of CGS can replace part of Portland cement, and the mechanical properties of coal gasification slag–cement composite cementitious materials are similar to PC 32.5 cement. Therefore, stimulating the cementitious activity of CGS to replace part of Portland cement has practical significance in environmental and economic aspects. The activation methods of auxiliary cementitious materials include mechanical grinding, chemical excitation and thermal activation. Single or composite excitation methods are often used for materials with potential cementitious activity. Mechanical grinding is one of the most commonly used methods to improve the performance of cementitious materials. With the increase in mechanical grinding degree, the fineness and SSA of cementitious materials also increase, but there is a limited degree of mechanical grinding. Excessive grinding may not improve the basic performance of cementitious materials but also increases the energy consumption cost.

Guo et al. [12] and WU et al. [13] have carried out basic research on the mechanical grinding and activation of CGS, but the influence of particle size distribution (PSD) characteristics of CGS on the mechanical properties of cemented specimens has not been expounded. Scholars at home and abroad for glass powder, fly ash, slag and other auxiliary cementitious materials research shows that the PSD of cementitious materials has a significant impact on the degree of hydration and the development of mechanical properties of cemented specimens. The influence of the particle number of different particle size groups on the early and late mechanical properties of cemented specimens is different [14,15,16,17,18,19,20,21]. Liu et al. [22] results show that when mechanically ground waste glass is used as an auxiliary cementitious material, 0–0.3 μm and 3–10 μm particles have significant effects on the early and late mechanical properties of cemented specimens, respectively. Zhou et al. [23] and Zhang et al. [24] also studied the correlation between PSD characteristics and cementitious activity of slag and fly ash.

The activation degree of CGS is very important for its development as an auxiliary cementitious material. In the present study, the CGS was mechanically grinded by planetary ball mill. The changes of physical and chemical characteristics of CGS were investigated by laser particle size analyzer, specific surface area analyzer, scanning electron microscope and infrared spectrometer. Based on the Divas–Aliavden grinding kinetics and RRB distribution model, the PSD characteristics of CGS at different grinding times were fitted, and the changes of particles in the grinding process of CGS were quantified. Finally, the influence of mechanical grinding on strength activity index of CGS was characterized by the mortar test, and the correlation analysis between PSD characteristics and strength activity index of CGS was carried out based on grey correlation theory.

## 2. Materials and Methods

### 2.1. Raw Materials

**Figure 1:**
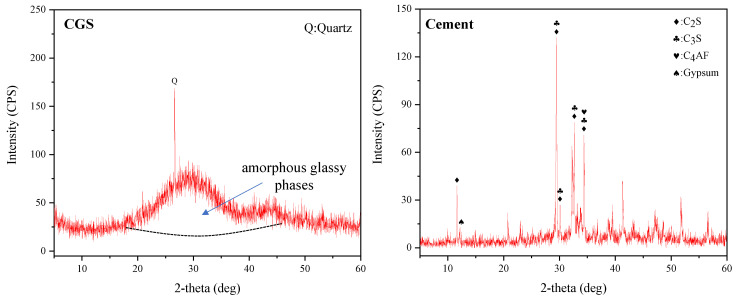
Mineral composition of CGS powder and cement.

The test materials include ISO standard sand (ISO), CGS and P.O 42.5 ordinary Portland cement. ISO standard sand is used as aggregate of mortar specimens, CGS and P.O 42.5 ordinary Portland cement are used as cementitious materials of mortar specimens. Due to the large amount of residual carbon in the fine fraction of CGS, the hydration reaction between CGS particles and Portland cement is hindered [25]. Therefore, auxiliary cementitious materials were prepared by coal gasification coarse slag. The coarse fraction of CGS originated from the gasifier of China Coal Group was dried, graded and ground to prepare the CGS powder, which can be used as an auxiliary cementing material. P.O 42.5 ordinary Portland cement is produced by Liquan Sea snail Cement Co., Ltd., Xianyang, China. Its performance indicators meet the requirements of the national standard “general Portland cement” (GB 175-2007). ISO standard sand is produced from Xiamen Aisiou Standard Sand Co., Ltd. (Xiamen, China), which meets the requirements of national standard for verification of standard sand for casting binder (GB/T 25138-2010). The silica content is more than 96%, the loss of ignition is less than 0.4%, and the mud content is less than 0.2%. The chemical composition and mineral composition of the test materials were measured by X-ray fluorescence spectrometer (XRF) and X-ray diffraction (XRD). The results are shown in Table 1 and Figure 1.

**Figure 2:**
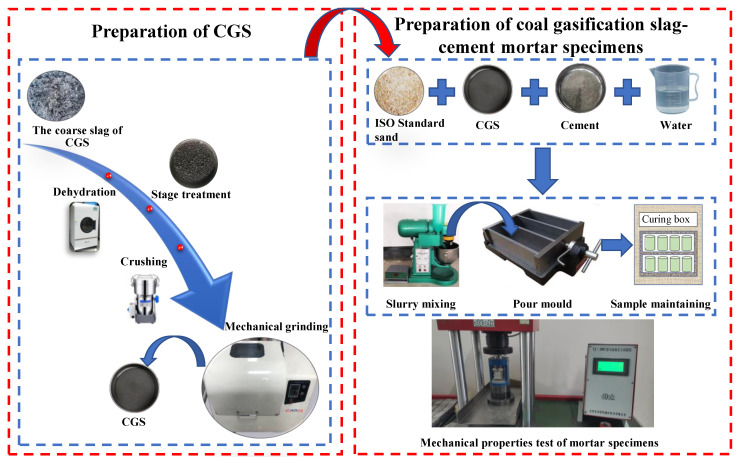
Preparation process of CGS powder and mortar sample.

The preparation process of CGS powder includes: (1) drying and dehydrating CGS using microwave dryer; (2) controlling the loss on ignition of CGS to be less than 8%, according to the requirements of the national standard “fly ash for cement and concrete” (GB/T 1596-2017); (3) crushing the dried coarse CGS with particle size greater than 1 cm to below 0.5 cm; (4) grinding the pre-crushed coarse CGS for 15, 30, 45, 60, 75 and 90 min using planetary ball mill. Finally, the CGS samples with different grinding time were prepared. The specific grinding parameters are as follows: the weight ratio of φ15, φ10 and φ5 mm steel balls in the planetary ball mill is 1:3:6 with total weight of 2 kg, and the rotation speed of the ball mill is 180 r/min. For each time, 600 g CGS sample is loaded for sealing grinding. The preparation process of CGS powder and mortar specimens is shown in Figure 2.

### 2.2. Test Methods

The PSD, SSA and microstructure characteristics of CGS with different grinding time were characterized by laser particle size analyzer (Malvern Mastersizer 2000, Malvern, UK), specific surface area analyzer (Mike 2020, Brisbane, Australia), scanning electron microscope (FEI Quanta 250 field emission, Lincoln, NE, USA) and infrared spectrometer (Nicolet iN10, Waltham, MA, USA). The measuring range of laser particle size analyzer is 0.1~1000 μm with dry detector. The acceleration voltage range of scanning electron microscope is 200 V–30 kV. The maximum beam current of the electron gun is 200 nA, and the magnification is 500× for microscopic image capture. The test wavelength of infrared spectrometer is 400–4000 cm^−1^.

According to the requirements of the national standard “cement mortar strength test method (ISO method)” (GB/T 17671-1999), the strength activity indexes of CGS mortar specimens with different grinding time for 3, 7 and 28 d were measured. The contents of CGS and cement are 30% and 70%, respectively. The water–cement and sand–binder ratios are 0.5 and 3, respectively. The size of the mortar test mold is 40 × 40 × 160 mm. Firstly, the test materials were weighed, stirred by mortar mixer for 3 min, and poured into the mortar test model. After curing in a room with controlled humidity and temperature for 48 h, the mold was removed, and then mortar samples were placed in a constant temperature and humidity curing box (temperature: 20 ± 1 °C, humidity: 95 ± 1%). Finally, the coal gasification slag-cement mortar specimens with curing ages of 3, 7 and 28 d were obtained. The uniaxial compressive strengths of coal gasification slag-cement mortar specimens at different curing ages were measured using MTS C43.504 universal testing machine. The intensity activity index is the ratio of coal gasification slag-cement mortar specimens and pure cement mortar specimens at the same curing age, as shown in Equation (1).

(1) $$H=\frac{UCS}{UCS_{0}}\times 100\%$$

where, *H* is the intensity activity index (%); *UCS* is the uniaxial compressive strength (MPa) of coal gasification slag-cement mortar specimen; *UCS*_0_ is the uniaxial compressive strength (MPa) of pure cement mortar specimens.

### 2.3. Mathematical Model

#### 2.3.1. Kinetic Equation of CGS Powder

Previous studies have revealed that the mechanical grinding process of most solid particles in the ball mill [21,25,26,27], including fly ash, furnace slag, waste glass powder, quartz, and other concrete admixtures, conforms to the Divas–Aliavden grinding kinetics equation. However, the effect of grindability and grinding time on particle characteristics of CGS as an auxiliary cementitious material has not been expounded. The mathematical expression of Divas–Aliavden grinding kinetic [21] Equation (2) is presented as follows:

(2) $$R=R_{0}e^{\left(-K_{t}\right)}t^{M}$$

where *R* is a sieve residue (by mass) of the ground powder material in a certain preset size; *R*_0_ is sieve residue of the material at zero grinding time in a certain preset size; *K_t_* is the grinding speed constant; *t* is grinding time; *M* is the time index, which is determined by the properties of grinding materials and grinding conditions. Once the parameters *K_t_* and *M* are determined, the kinetic equation can be established to describe the change of particle size in the grinding process of powder materials.

#### 2.3.2. RRB Model

Generally, cement clinker and concrete admixtures, including furnace slag, fly ash, and desulfurization gypsum, are ground together to produce cement powder. The PSD of ground materials is usually described by the RRB model quantificationally. The parameter particle size (d*) and uniformity coefficient (*n*) are the key parameters of the RRB model for ground powder materials. These two parameters quantify the grinding degree of powder materials and characterize the influence of PSD characteristics of powder materials on the performance of cemented specimens intuitively [28,29,30,31,32]. The mathematical expression of the RRB model is shown as Equation (3). Logarithms are taken on both sides of Equation (3) to realize the linear transformation in the form of “lnln-ln”, as shown in Equation (4).

(3) $$CSS=100\exp\left[-{\left(\frac{d}{d^{*}}\right)}^{n}\right]$$

(4) $$\ln\ln\left(\frac{100}{CSS}\right)=n\ln d-n\ln d^{*}$$

where *CSS* is the cumulative sieve residue (by mass) of powder material particles; *d* is the particle size of powder material; d* is the characteristic particle size of powder material particles (the particle size corresponding to sieve amount reaching 36.79%); *n* is the uniformity coefficient of PSD of powder materials. The larger the uniformity coefficient is, the more concentrated the PSD of powder materials is.

## 3. Results and Discussion

### 3.1. PSD and Microstructure of CGS with Different Grinding Time

#### 3.1.1. PSD Characteristics

**Figure 3:**
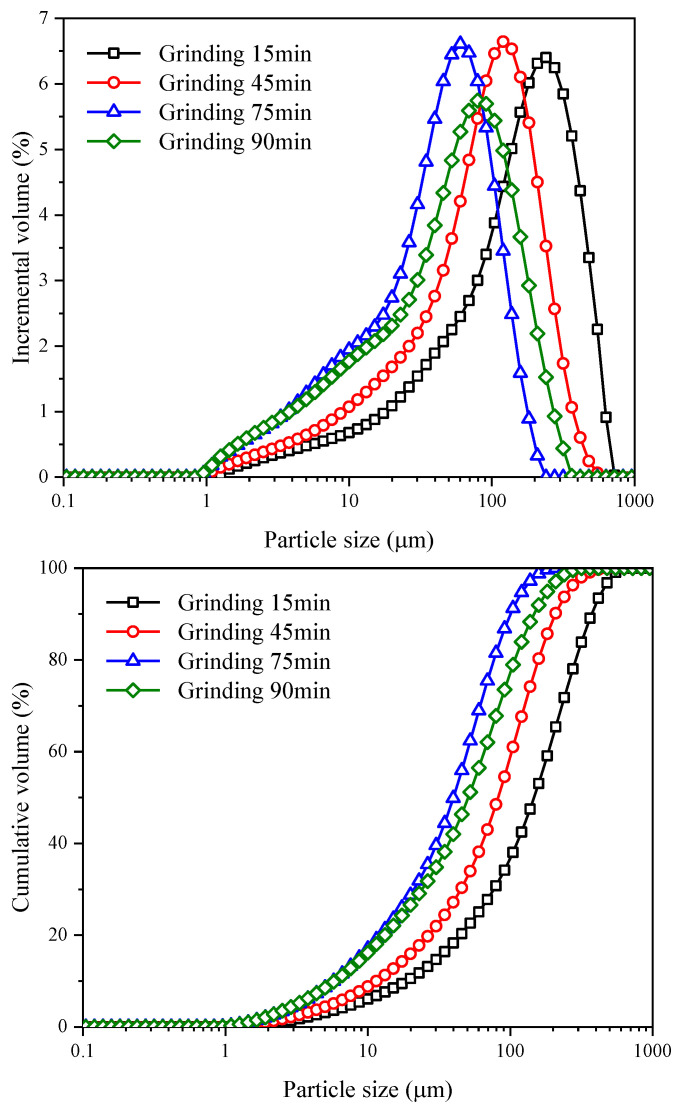
The PSD curve of CGS in different grinding time.

Figure 3 shows the frequency distribution and cumulative distribution curve of CGS particle size with different grinding time. The curves reveal that the mechanical grinding of CGS has an obvious influence on its PSD. The maximum particle size of CGS decreases from 724.44 μm to 208.93 μm, and the minimum particle size decreases from 1.26 μm to 0.95 μm with the increasing of grinding time in the range of 15–75 min This shows that the CGS particles are gradually refined, and the PSD is gradually concentrated with the increase in grinding time. However, the maximum particle size increased from 208.93 μm to 363.08 μm with the increasing of grinding time in the range of 75–90 min. This phenomenon indicates that mechanical grinding of CGS has a limitation on particle refinement, and excessive grinding will make particles larger.

#### 3.1.2. Microstructure Characteristics

**Figure 4:**
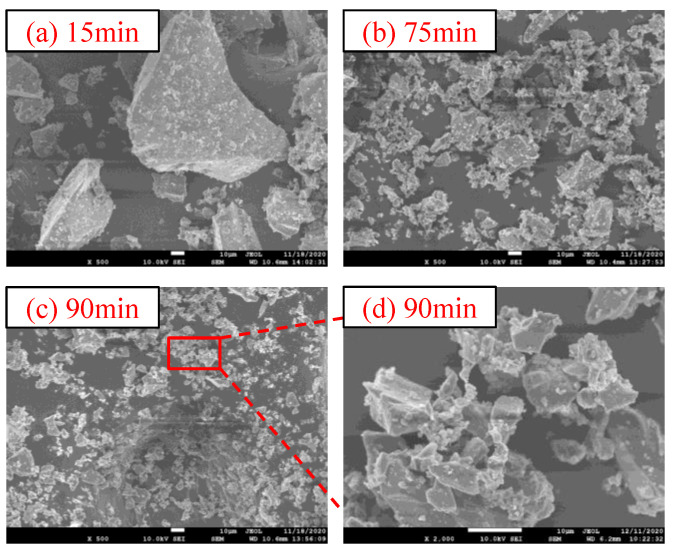
Microstructure characteristics of CGS with different grinding time.

Figure 4 shows the microstructure characteristics of CGS with different grinding time. It shows that the ungrounded CGS is mainly composed of massive particles. With the increase in grinding time, the massive particles gradually decrease, and the detrital particles gradually increase, and the grinding roundness of the particles is gradually improved. The microscopic morphology of the CGS after mechanical grinding for 90 min is partially enlarged to 2000× for observation. The “agglomeration phenomenon” [33,34] of the detrital particles is obviously observed in Figure 4. By comparing the micromorphology of CGS powders with different grinding time, the agglomeration phenomenon of CGS powders ground 90 min was analyzed. The mechanical grinding of CGS reduces the bond energy of the Si–O bond, resulting in a decrease in the crystallinity degree [35]. Meanwhile, the surface free energy of CGS particles tends to increase with the increase in mechanical grinding time. This property promotes the peripheral particle to absorb a certain amount of water from the surrounding environment, forming hydroxyl layer and multilayer physically adsorbed water on the surface [36]. The mechanical grinding process of powder materials can be divided into three stages, that is, particle stress refinement stage, stable stage and agglomeration stage:

(1) Particle stress refinement stage: Due to the high-speed operation of the grinding ball in the ball mill, the CGS particles are subjected to the collision stress of the grinding ball, and the particles are cracked and refined under impact. At this stage, the SSA of the CGS is linear with the grinding time. Since the whole process belongs to sealed grinding, a large amount of internal energy is generated during the operation of the system, which leads to the decline of the crystallization degree of the CGS particle crystal, the decrease in the bond energy of the internal chemical bond and the gradual increase in the surface-free energy in the system.
(2) Stable stage: In this stage, the CGS gradually tends to the grinding limit, and the crystallization degree, chemical bond energy and surface free energy of CGS particles gradually tend to be stable.
(3) Agglomeration stage: At this stage, due to the high surface free energy of CGS particles, they are more active and absorb a certain amount of water from the surrounding environment, forming a hydroxyl layer on the surface. The formation of hydroxyl layer reduces the electrostatic repulsion effect due to the relaxation phenomenon on the particle surface, and the formation of van der Waals force and hydrogen bond between hydroxyl groups leads to the agglomeration of CGS particles. After that, continuing the mechanical grinding of CGS leads the particle state gradually into reversible equilibrium.

### 3.2. Grinding Kinetics and RRB Model of CGS

**Figure 5:**
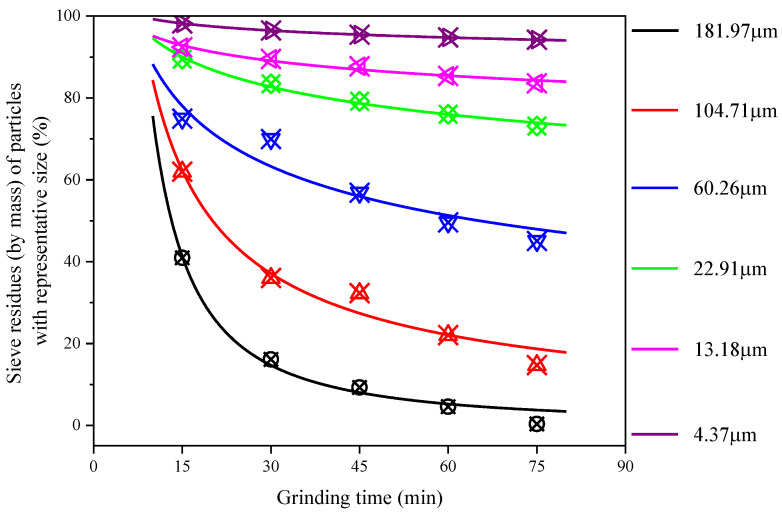
Fitting curve of sieve allowance and grinding time of representative particle size of CGS.

According to the Divas–Aliavden grinding kinetics equation and cumulative distribution curve of CGS, six representative particle sizes (4.37 μm, 13.18 μm, 22.91 μm, 60.26 μm, 104.71 μm and 181.97 μm) were selected, and six representative particle sizes were calculated, as shown in Table 2. According to the equation fitting the relationship between the sieve amount of six representative particle sizes and grinding time, the PSD of CGS grinding for 90 min is complex and difficult to control due to the “agglomeration phenomenon”, so the grinding kinetics and RRB model are not fitted. The fitting curves and parameters of grinding kinetics are shown in Figure 5 and Table 3.

It can be seen from the fitting results of grinding kinetics that the Divas–Aliavden grinding kinetic equation can highly describe the change of particles in the grinding process of CGS and quantitatively characterize the particles with different sizes in the grinding process. All fitting curves showed a downward trend with the extension of grinding time. After 75 min grinding, all curves gradually stabilized, and the larger the representative particle size, the more obvious the downward trend. It further shows that the grinding efficiency of coarse particles is higher, and the grinding efficiency gradually tends to zero after 75 min grinding.

**Figure 6:**
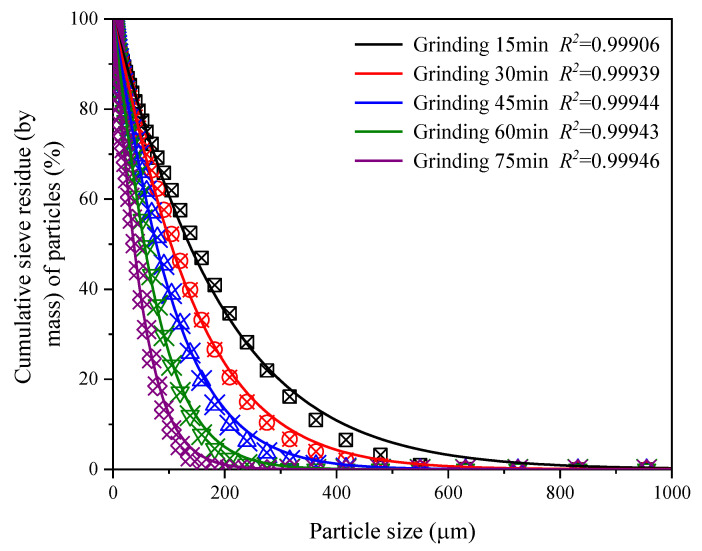
Fitting curve of RRB distribution model of CGS with different grinding time.

According to the RRB distribution model, the measured data of PSD of CGS at different grinding times are fitted. The fitting curves and fitting parameters are shown in Figure 6 and Table 4, respectively. It can be seen from Figure 6 and Table 4 that the RRB distribution model can better characterize the PSD characteristics of CGS with different grinding time, and the fitting value of the parameter particle size d* is close to the measured value, and the error is less than 5%. With the increase in grinding time, the characteristic particle size d* gradually decreases, and the distribution index *n* gradually increases. This shows that with the increase in grinding time, the CGS particles are gradually refined and the PSD is gradually concentrated. The distribution index *n* is closely related to the mechanical properties of the cemented specimen. Improving the PSD characteristics of the cementing material is conducive to improving the accumulation degree of particles in the cemented specimen, reducing the porosity and improving the compactness of the cemented specimen [25].

### 3.3. Characteristic Particle Size and SSA of CGS with Different Grinding Time

**Figure 7:**
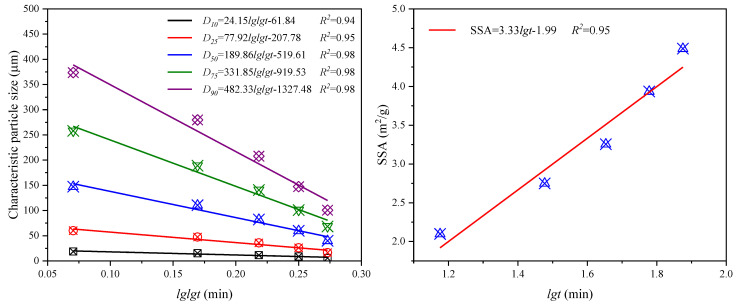
Relationship between characteristic particle size, SSA and grinding time of CGS.

In this study, the characteristic particle size was introduced to characterize the effect of grinding time on the PSD of CGS. The characteristic particle size was D_10_, D_25_, D_50_, D_75_ and D_90_ (D_10_ indicates that the particle size of CGS less than D_10_ accounts for 10% of the total). Table 5 gives the characteristic particle size and SSA of CGS with different grinding time. Figure 7 are the relationship between the characteristic particle size and SSA of CGS and grinding time.

It can be seen from Table 5 that with the increase in grinding time, the characteristic particle size decreases gradually and SSA increases gradually. This shows that with the increase in grinding time, the content of coarse particles that have a significant impact on the characteristic particle size decreases gradually, and the content of fine particles and debris increases gradually, which promotes the increase in SSA of CGS. It can be seen from Figure 7 that the characteristic particle size and SSA of CGS have a good linear relationship with the logarithm of grinding time, respectively, and the correlation degree is high. The fitting equation can quantitatively characterize the change of particle size and SSA during the grinding process of CGS. With the increase in grinding time, the larger the characteristic particle size, the faster the decline rate, which further illustrates that the grinding efficiency of coarse particles is relatively large.

### 3.4. Particle Size Fractal Dimension of CGS with Different Grinding Time

Domestic and foreign scholars believe that there are many factors affecting the macroscopic properties of cement-based materials, and the particle swarm characteristics of raw materials belong to one of many factors, including particle size, distribution range, particle shape and other parameters. Yu et al. [35] used fractal theory to analyze the PSD of powders and pointed out that fractal dimension could better characterize the PSD characteristics of powders. Fractal theory can scientifically reflect the uniformity and dispersion trend of material particles, and it is gradually widely used in describing the PSD characteristics of brittle materials [15,18,37,38]. Therefore, this study preliminarily believes that the PSD of CGS has fractal characteristics in the grinding process and calculates the fractal dimension of PSD of CGS with different grinding time according to Equation (5) [39].

(5) D=3−b

where *D* is the fractal dimension of PSD of powder material; *b* is the slope of lg[m(*d*)/m] − lg*d* curve; m(*d*)/m is the cumulative mass fraction of particle size *d* of powder material; *d* is the particle size of powder material.

**Figure 8:**
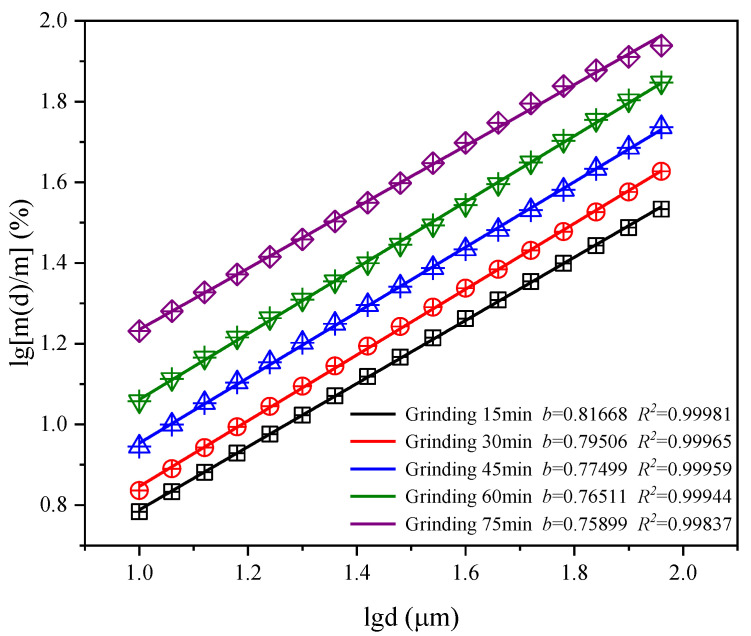
lg[m(*d*)/m] − lg*d* curves.

**Figure 9:**
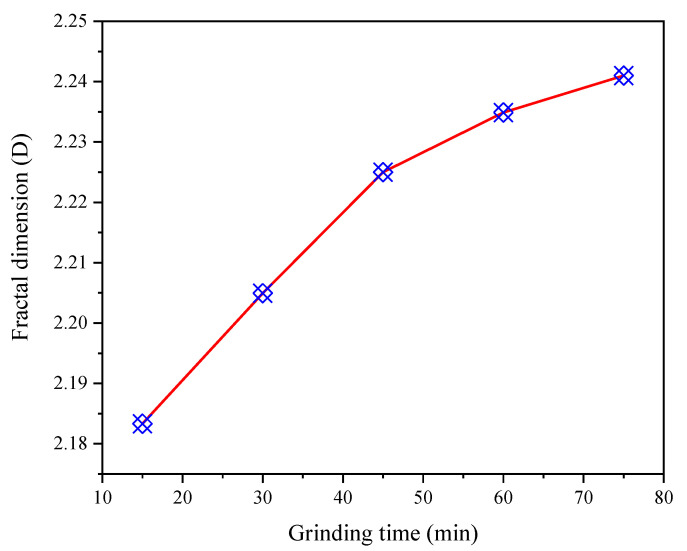
Relationship between grinding time and fractal dimension.

Figure 8 is the fitting curve of lg[m(*d*)/m] − lg*d* of CGS under different grinding time. The graph shows that the lg[m(*d*)/m] − lg*d* of CGS has obvious linear relationship under different grinding time, and the correlation coefficient is high. Therefore, the PSD of CGS with different grinding time has fractal characteristics. It can be seen from Figure 9 that with the increase in grinding time, the fractal dimension of the PSD of CGS gradually increases. After grinding for more than 45 min, the increase rate of the fractal dimension of the PSD of CGS slows down. The larger the fractal dimension is, the more difficult the particle of CGS is to be broken. At the beginning of grinding, the CGS particles are large, and under the action of external mechanical force, the particles are mainly broken by volume, and the particles are gradually refined. With the continuous extension of grinding time, the volume breakage, mainly due to collision, extrusion and shearing, is gradually weakened. At the later stage of grinding, the particles of CGS are refined to the limit, the surface-free energy is gradually increased and each other are adsorbed, and the “agglomeration phenomenon” gradually occurs.

**Figure 10:**
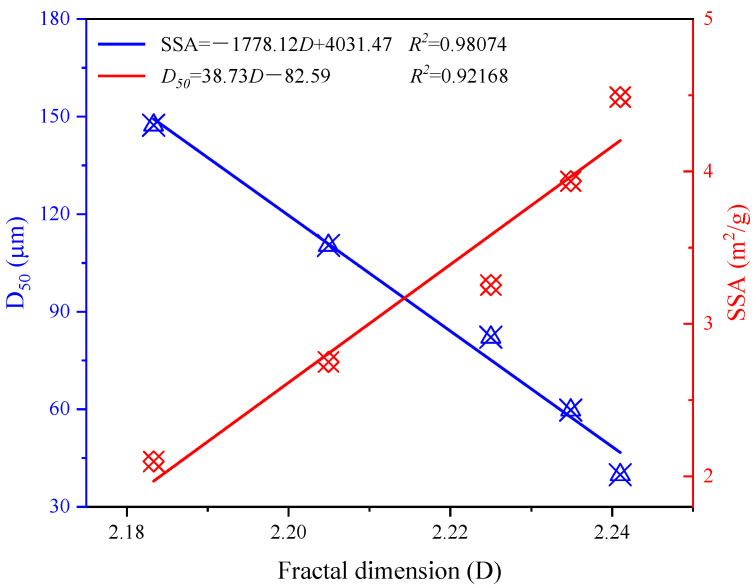
Relationship between fractal dimension and SSA, D_50_ of CGS.

Figure 10 shows the relationship between fractal dimension and SSA, D_50_ of CGS. It can be seen from the figure that the fractal dimension of the PSD of CGS has a good linear relationship with the SSA and D_50_, and the correlation coefficient is high. The fractal dimension reflects the dispersion degree of CGS particles after ball milling; at the same time, the SSA is related to the characteristics of PSD and particle morphology of CGS. Therefore, the fractal dimension of PSD of CGS is basically consistent with the characteristics of CGS characterized by SSA.

### 3.5. Strength Activity Index of CGS with Different Grinding Time

**Figure 11:**
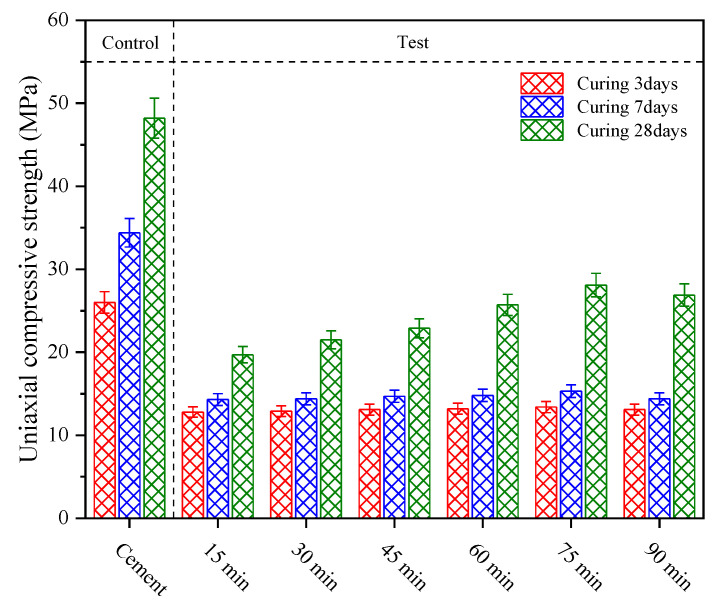
Uniaxial compressive strength test results of coal gasification slag-cement mortar at different grinding time.

Figure 11 shows the strength test results of coal gasification slag-cement mortar with different grinding time. It can be seen from the figure that the uniaxial compressive strength of pure cement mortar specimens in the control group after 28 d of curing is 48.2 MPa, and that of CGS mortar specimens in the experimental group after 28 d of curing is 19.7 MPa, 21.5 MPa, 22.9 MPa, 25.7 MPa, 28.1 MPa and 26.9 MPa, respectively. With the increase in grinding time, the performance of CGS particles in the mortar specimens was gradually improved, and the uniaxial compressive strength of the specimens was gradually increased. However, the uniaxial compressive strength of the mortar specimens grinded with CGS for 90 min showed a decrease, which was closely related to the microstructure characteristics of CGS after mechanical grinding for 90 min. It can be attributed to the weakening of the micro-aggregate effect and volcanic ash effect of CGS particles in the mortar specimens.

**Figure 12:**
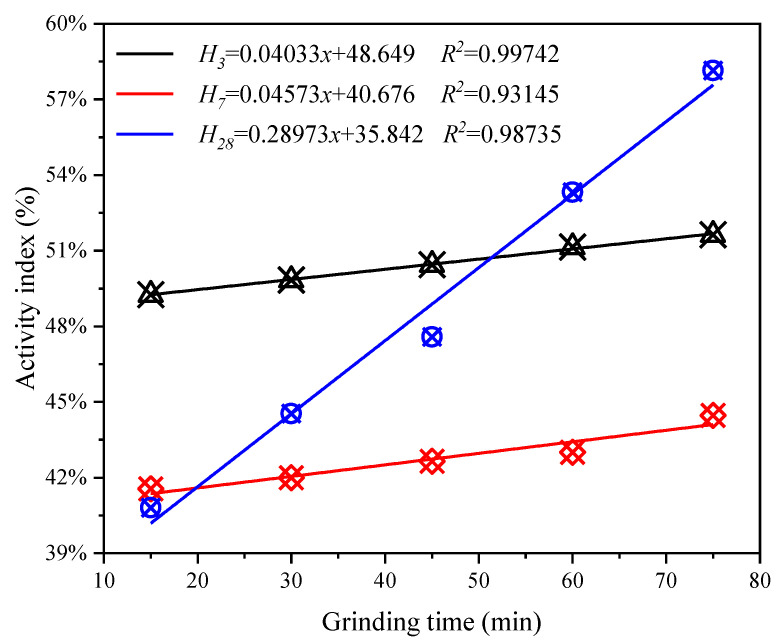
Relationship between activity index of CGS and grinding time.

Based on the uniaxial compressive strength test results of coal gasification slag-cement mortar specimens, the strength activity index of CGS at different grinding times was calculated according to Formula (1). Figure 12 shows the relationship between the strength activity index of CGS and the grinding time. It can be seen from the figure that, among the linear fitting slopes of the strength activity index of CGS with different grinding time, the curing time of 28 d is the largest, followed by the curing time of 7 d and 3 d. The results show that mechanical grinding has a remarkable effect on the uniaxial compressive strength of coal gasification slag-cement mortar specimens, especially on the later mechanical properties of CGS.

Previous studies have shown that the particle size gradation of Portland cement has a significant effect on its mechanical properties [40,41,42]. Among them, the number of particles in the range of 0–3 μm and 3–24 μm has a significant impact on the mechanical properties of the cured samples for 1 d and 28 d, respectively. Therefore, based on SPSS software, grey correlation analysis was used to explore the influence of PSD group of CGS on its strength activity index. Taking the particle group of CGS as the subsequence and the strength activity index of curing 3 d, 7 d and 28 d as the parent sequence, the grey correlation degree between the particle group of CGS and the strength activity index is calculated. The results are shown in Table 6.

Table 6 reveals that the CGS powder with particle size ranging in 20–30 μm has the greatest influence on the strength activity index of mortar specimens cured for 3 d and 7 d, and the CGS powder with particle size ranging in 10–20 μm interval has the greatest influence on the strength activity index of mortar specimens cured for 28 d. Compared with the size of CGS particle group, which plays a leading role in the early and late mechanical properties of mortar specimens, the development of mechanical properties needs a relatively small particle group. This shows that the early hydration time of cemented specimens is relatively short, and the crystal development of hydration products is not stable. The development of early mechanical properties mainly depends on the accumulation degree between particles, and the system has a good gradation, which has a strong promoting effect on the early mechanical properties. Relatively small particles can participate in the hydration reaction to a large extent, so that more hydration products are produced inside the specimen, filling the internal pores of the specimen and cementing the inert particles. The degree of hydration and the number of hydration products have a significant impact on the later mechanical properties. Therefore, the change of early mechanical properties of mortar specimens is mainly affected by the micro-aggregate effect of CGS, and the volcanic ash effect is supplemented. The volcanic ash effect of CGS is the main factor affecting the change of mechanical properties of mortar specimens in the later stage, supplemented by the micro-aggregate effect.

## 4. Conclusions

(1) The results of PSD and microstructure of CGS by mechanical grinding show that with the increase in grinding time, the PSD of CGS is gradually concentrated, and the internal Si–O bond and water molecular structure are gradually destroyed. When the grinding time is more than 75 min, the hydroxyl layer will be formed on the surface of CGS particles. Van der Waals force and the hydrogen bond between hydroxyl groups will lead to “agglomeration phenomenon” of particles.
(2) The mechanical grinding process of CGS can be quantitatively described by Divas–Aliavden grinding kinetics. The grinding efficiency of coarse particles is relatively high, and with the increase in grinding time, the grinding efficiency of coarse and fine particles gradually decreases and tends to zero. The PSD of CGS has a strong correlation with RRB model. With the increase in grinding time, the parameter particle size d* decreases and the distribution index *n* increases, indicating that the PSD of CGS tends to be concentrated.
(3) With the increase in grinding time, the characteristic particle size of CGS gradually decreases, and the SSA gradually increases. Moreover, the characteristic particle size and SSA of CGS have a good linear relationship with the double logarithm and logarithm of the grinding time, respectively.
(4) The PSD of CGS has obvious fractal characteristics. With the increase in grinding time, the fractal dimension of CGS particles increases gradually, which increases the difficulty of grinding. The fractal dimension has a good linear positive correlation with D_50_ and a good linear negative correlation with SSA.
(5) The grinding time has a linear positive correlation with the strength activity index at different curing ages, which mainly affects the later strength activity index. The number of CGS particles at 20–30 μm and 10–20 μm has the greatest impact on the early and late strength activity indexes, respectively. Therefore, considering the economic and technical aspects, it is recommended that the optimal mechanical grinding time of CGS is 75 min.

## Figures and Tables

**Table 1:** Chemical composition of the test material.

Testing Material	Chemical Composition (%)										
	SiO_2_	Al_2_O_3_	Fe_2_O_3_	Na_2_O	CaO	SrO	SO_3_	K_2_O	MgO	TiO_2_	Other
ISO standard sand	+96	-	-	-	-	-	-	-	-	-	−4
CGS	30.94	11.01	23.22	5.58	17.19	3.28	2.02	0.78	0.83	0.61	4.54
Cement	19.19	4.50	3.33	0.18	64.13	0.03	2.40	0.40	1.82	0.20	3.82

**Table 2:** The sieve allowance of six representative particle sizes of CGS.

Representative Particle Size	15 min	30 min	45 min	60 min	75 min
181.97 μm	40.93	16.1	9.27	4.55	0.33
104.71 μm	61.97	36.02	32.29	22.06	14.74
60.26 μm	74.94	69.94	56.83	49.58	45.03
22.91 μm	89.48	83.45	79.18	76.01	73.16
13.18 μm	92.39	89.53	87.71	85.37	83.59
4.37 μm	98.22	96.49	95.44	94.76	94.23

**Table 3:** Grinding kinetic equation of representative particle size of CGS.

Representative Particle Size/μm	Kinetic Equation	R^2^
181.97	$R=R_{0}e^{7.75997}t^{-1.49166}$	0.98463
104.71	$R=R_{0}e^{6.15707}t^{-0.74787}$	0.96857
60.26	$R=R_{0}e^{5.17794}t^{-0.30297}$	0.89987
22.91	$R=R_{0}e^{4.83151}t^{-0.12242}$	0.98981
13.18	$R=R_{0}e^{4.69407}t^{-0.06016}$	0.96745
4.37	$R=R_{0}e^{4.65723}t^{-0.02586}$	0.99988

**Table 4:** Fitting parameters of RRB distribution model of CGS with different grinding time.

Grinding Time/min	*n*	d*/μm		
		Fitting Values	Measured Values	Error/%
15	1.08	191.31	199.49	4.1
30	1.11	143.77	147.51	2.5
45	1.12	107.26	109.66	2.2
60	1.15	77.04	78.88	2.3
75	1.17	51.73	53.12	2.6

**Table 5:** Characteristic particle size and SSA of CGS at different grinding time.

Characteristic Particle Size	15 min	30 min	45 min	60 min	75 min
D_10_/μm	18.72	15.31	11.52	8.76	5.78
D_25_/μm	59.99	47.44	35.77	26.08	16.45
D_50_/μm	147.37	110.38	82.12	59.72	39.87
D_75_/μm	257.99	188.92	140.86	100.85	68.39
D_90_/μm	373.61	279.84	207.71	147.37	100.62
SSA/m^2^·g^−1^	2.0973	2.7501	3.2548	3.9356	4.4874

**Table 6:** Grey correlation between particle group of CGS and strength activity index.

Curing Age/Day	Particle Size Range/μm					
	0–3	3–10	10–20	20–30	30–60	+60
3	0.597	0.578	0.603	0.622	0.546	0.576
7	0.606	0.587	0.614	0.632	0.555	0.587
28	0.632	0.660	0.706	0.680	0.630	0.503

## Data Availability

Not applicable.

## Abbreviations

The abbreviation in this paper include: particle size distribution (PSD); coal gasification slag (CGS); Rosin–Rammler–Bennet (RRB); specific surface area (SSA); X-ray fluorescence spectrometer (XRF); X-ray diffraction (XRD).
